# PDRLGB: precise DNA-binding residue prediction using a light gradient boosting machine

**DOI:** 10.1186/s12859-018-2527-1

**Published:** 2018-12-31

**Authors:** Lei Deng, Juan Pan, Xiaojie Xu, Wenyi Yang, Chuyao Liu, Hui Liu

**Affiliations:** 1 School of Software, Central South University, Changsha, 410075 China; 2 Lab of Information Management, Changzhou University, Changzhou, 213164 China

**Keywords:** DNA-binding residue, Light gradient boosting, Random forest, Incremental feature selection

## Abstract

**Background:**

Identifying specific residues for protein-DNA interactions are of considerable importance to better recognize the binding mechanism of protein-DNA complexes. Despite the fact that many computational DNA-binding residue prediction approaches have been developed, there is still significant room for improvement concerning overall performance and availability.

**Results:**

Here, we present an efficient approach termed PDRLGB that uses a light gradient boosting machine (LightGBM) to predict binding residues in protein-DNA complexes. Initially, we extract a wide variety of 913 sequence and structure features with a sliding window of 11. Then, we apply the random forest algorithm to sort the features in descending order of importance and obtain the optimal subset of features using incremental feature selection. Based on the selected feature set, we use a light gradient boosting machine to build the prediction model for DNA-binding residues. Our PDRLGB method shows better overall predictive accuracy and relatively less training time than other widely used machine learning (ML) methods such as random forest (RF), Adaboost and support vector machine (SVM). We further compare PDRLGB with various existing approaches on the independent test datasets and show improvement in results over the existing state-of-the-art approaches.

**Conclusions:**

PDRLGB is an efficient approach to predict specific residues for protein-DNA interactions.

## Introduction

Th protein-DNA interaction is one of the central issues in molecular biology and widely exists in various biological activities in living organisms, such as DNA replication, repair, and modification processes. To understand the recognition mechanism of protein-DNA complexes, researchers often focus on protein-DNA binding sites especially the interface residues that bind DNA. Experimental approach such as electrophoretic mobility shift assays (EMSAs) [1, 2], conventional chromatin immunoprecipitation (ChIP) [3], X-ray crystallography [4], PNA (peptide nucleic acid)-assisted identification of RNA binding proteins (PAIR) [5], and NMR spectroscopy [6] have been applied to expose the DNA binding amino acids. However, these laboratory methods are expensive and time-consuming. Alternatively, low-cost and efficient computational methods are particularly important in discovering specific interface residues of protein-DNA complexes.

A number of computational approaches have been focused on applying machine learning algorithms to build prediction models based on sequence and structural information. Wei [7] proposed novel evolutionary features for DNA-binding proteins prediction. Jones and his coworkers [8] proposed a simple method to identify DNA-binding residues using the positive electrostatic patches on the protein surface. Ahmad et al. [9] developed a neural network classifier to predict DNA-binding residues using a variety of composition, sequence and structural information. Wang et al. [10] built SVM-based models to predict DNA-binding residues by using data examples represented with three sequence characteristics. Ferrer-Costa et al. [11] implemented an effective linear predictor to determine the DNA-binding sites in protein sequences. Yan and his coworkers [12] trained a Naive Bayes classifier to predict whether a given amino acid is a DNA-binding site based on its characteristics and the features of its sequence neighbors. Wang and Yang [13] developed a random forest (RF) classifier according to the evolutionary information to detect the DNA-binding sites. Song et al. [14] employed imbalanced classification techniques for this problem. Carson et al. [15] combined the C4.5 algorithm with bootstrap aggregation and cost-sensitive learning to identify binding residues in protein-RNA complexes. Zou et al. [16] focused on the feature selection techniques and improved the performance. Ozbek et al. [17] presented a prediction method based on residue variations in high frequency forms using the Gaussian network. Other protein-DNA binding residue prediction tools such as DR_bind [18] and PreDNA [19] have also been developed.

Although a lot of studies has been performed, the problem of accurately identifying protein-DNA binding sites still has huge room for improvement. Firstly, effective features to detect DNA-binding interface residues from non-binding amino acids are not fully exploited. Secondly, the imbalanced problem exists since the numbers of DNA-binding and non-binding amino acids in proteins are extremely unbalanced, and will cause over-fitting and poor performance in the prediction of DNA-binding amino acids.

In this work, we develop a innovative computational pipeline, named PDRLGB, for predicting interface residues in protein-DNA complexes. We extract many sequence and structure features and use the random forest to select a subset of optimal features. Based on the selected characteristics, we train the DNA-binding residue prediction models using a new implementation of Gradient boosting decision tree (LightGBM) [20]. Our experiments show that PDRLGB significantly outperforms other state-of-the-art DNA-binding residue prediction approaches.

## Materials and methods

### Datasets

To access the performance of the PDRLGB method and other existing approaches, two benchmarking datasets (PDNA-62 and PDNA-224) and two independent datasets (TS-72 and TS-61) are used. PDNA-62 was built by Ahmad et al. [9]. It consists of 67 sequences obtained from 62 protein-DNA complexes in the Protein Data Bank (PDB) [21] and the sequence identity between any two sequences is ≤ 25%. PDNA-224 was generated by Li et al. [19], which contains 224 proteins and the redundant sequences was removed by using the sequence identity cutoff of 25%. The independent test dataset called TS-72 was extracted by Ma et al. [22]. It contains 72 protein chains. TS-61 was constructed by Zhou et al. [23]. Redundant proteins are removed by using the CD-HIT [24], and the remaining 61 non-redundant DNA-binding protein sequences have ≤30% sequence identity with the protein sequences in PDNA-62, PDNA-224, and TS-72.

Similar to previous researches [10, 18], a residue of a protein is defined as a binding amino acid if the closest distance between atoms of the protein and its binding DNA is ≤3.5Å. The whole positive samples and negative samples of the four datasets are summarized in Table 1.

**Table 1:** Number of positive samples (binding sites) and negative samples (non-binding sites) of the four datasets

Dataset	Positive samples	Negative samples
PDNA-62	1215	6948
PDNA-224	3778	53,570
TS-72	1040	13,226
TS-61	1078	13,175

### Performance measures

To evaluate the performance, we use several typical measures, including accuracy (ACC), sensitivity (SN/Recall), specificity (SP), strength (ST), precision (PRE), F1-score (F1), and Matthews Correlation Coefficient (MCC) score. These measurements are defined as:

1 $$ ACC = \frac { TP + TN }{ TP + TN + FP + FN }  $$

2 $$ SN=TP/(TP+FN)  $$

3 $$ SP=TN/(TN+FP)  $$

4 $$ ST= (SN+SP)/2  $$

5 $$ PRE = TP/(TP+FP)  $$

6 $$ F1 = \frac { 2 \times SN \times PRE }{ SN + PRE }  $$

7 $$ MCC \,=\, \frac{TP \times TN - FP \times FN}{ \sqrt {(TP \,+\, FP)(TP + FN)(TN + FP)(TN+FN)}}  $$

In these equations, the TP, FP, TN, and FN represent the number of true positives, the number of false positives, the number of true negatives, and the number of false negatives, respectively. Because of the imbalanced problem in the data sets, the strength (ST) is the average score of sensitivity and specificity which is used to obtain a fair measure of the model. Additionally, there are two broadly employed measurement to estimate prediction performance including the receiver operating characteristic (ROC) [25] and the area under ROC curve (AUC) [26]. The ROC curve is plotted with the false positive rate against the true positive rate. When AUC takes the maximum value of 1, it represents a perfect predictor, and the values of AUC of random guessing is usually close to 0.5.

### The prediction pipeline

**Fig. 1:**
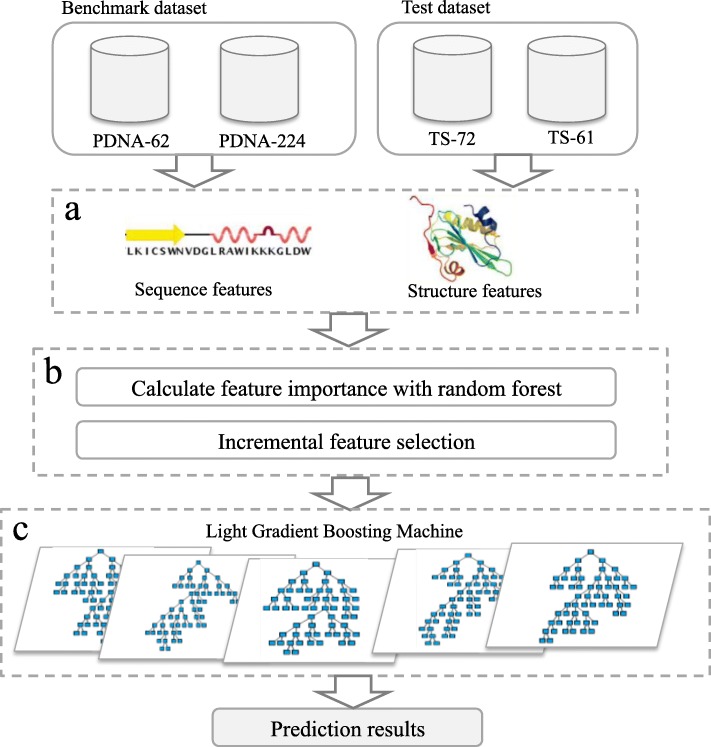
Flowchart of PDRLGB. It includes three steps: **a** extract a variety of sequence and structure features; **b** apply the random forest algorithm to sort the features in descending order and select a subset of essential features using the incremental feature selection approach; **c** build DNA-binding residue prediction models using LightGBM

The pipeline of PDRLGB is showed in Fig. 1. It is made up of several steps: A) feature extraction: a total of 83 sequence and structure features are extracted, and the feature vectors are generated using a sliding window of *w*; B) feature selection: the features are sorted with random forest and the optimal feature set is selected using the incremental feature selection approach; C) building prediction classifiers: the DNA-binding residue prediction models are built using the light gradient boosting machine. These processes are described in details in the following subsections.

### Feature extraction

We extract a variety of features including position-specific scoring matrices (PSSMs) (20 features), physicochemical properties (10 features), disordered features (3 features), side-chain environment (pKa) (2 features), identity vector (20 features), net charge (1 feature), the information from DSSP (15 features), the information from NACCESS (10 features), H-bonds (1 feature) and B-factor(1 feature). These features can be grouped into two categories: sequence and structure features.

Sequence features:

1. Position-specific scoring matrices (PSSMs): PSSM based evolutionary information is obtained from multiple sequence alignment calculated by PSI-BLAST [27] searching against the NCBI non-redundant (NR) database, with iteration number as 3 and e-value as 0.001.
2. Physicochemical properties: The physicochemical properties of a residue include atom numbers, electrostatic charge numbers, potential hydrogen bonds, molecular mass (Mmass), hydrophobicity, hydrophilicity, polarity, polarizability, propensities and average accessible surface area [28]. The original values of the ten physicochemical attributes for each residue are obtained from the AAindex database [29].
3. Disordered regions: Predicted disordered regions within a protein is also a significant property. Avoiding possibly disordered fragments in protein expression constructs can enhance expression, foldability, and stability of the protein. DisEMBL [30] is a useful tool for identifying disordered regions, which is needed for many biochemical studies, particularly structural biology, and structural genomics projects. In this study, DisEMBL is used to indentify dynamically disordered regions of the protein sequence.
4. Side-chain environment (pKa): The value of pKa is an effective metric in determining environmental features of a protein. The side-chain pKa rates are collected from Nelson and Cox [31] representing protein side-chain environmental factors and are broadly used by previous studies.
5. Identity vector: There is a 20-feature vector with 1 when the residue type occurs at the corresponding position and 0 for the remaining amino acid types.
6. Net charge of a residue: Twenty amino acids can be divided into non-polar amino acids, polar charged amino acids, polar uncharged amino acids. The DNA backbone is negatively charged, so the sequence of polar positively charged amino acids is thought to be characteristic of DNA binding. A charge of +1 is assigned to Arg and Lys and -1 to Asp and Glu. His is assigned a charge of +0.5 and all other residues are regarded as neutral.

Structure features:

1. Features from DSSP: we use DSSP [32] to obtain the secondary structures, including solvent accessible surface area (ASA), hydrogen bonds, atom coordinates and backbone torsion angles.
2. Features from NACCESS: We use NACCESS [33] to compute the absolute and relative ASA of all atoms, total side chain, main chain, non-polar side chain and allpolar side chain, respectively. ASA related features has been shown to be a important feature in identifying protein functional sites [34–37].
3. Number of H-bonds: The number of Hydrogen bonds (Hbond) is computed by HBPLUS [38].
4. B-factor of a residue: The B-factor [39] of protein crystal structures, including the B-factor of the *C*_*α*_ and that of the *C*_*β*_ of the amino acids in the sequence, was adopted.

### Features selection

We encode the features with a sliding window of *w* and generate a large feature vector. To eliminate uninformative variables and obtain more cost-effective models, a reliable feature selection approach was applied. Firstly, we use the random forest algorithm [40] to sort the features by using the mean decrease Gini index (MDGI) Z-Score [41]. MDGI Z-Score measures the importance of individual features. Features with higher MDGI Z-Scores are more sensitive to random shuffling of their values, and thus are more important for correctly classifying a residue into DNA-binding site and non-DNA binding site. After ranking the features in descending order of MDGI Z-Score, we utilize the incremental feature selection approach to select the top-*k* features. We construct the feature subset by incremental adding the features in the ranked list to the subset, and evaluate the performance of the top-*k* subset using the LightGBM classifier with 5-fold cross-validation. We use a comprehensive evaluation score (*R*_*c*_) to measure the performance of the feature subset. The *R*_*c*_ score is defined as follows:

8 $$ R_{c}=\frac{1}{n}\sum\limits_{i=1}^{n}\left\{{ACC}_{i}+{SN}_{i}+{SP}_{i}+{AUC}_{i}\right\},  $$

where *n* is the repeat times of the 5-fold cross-validation.

### Building prediction classifiers

Gradient boosting decision tree (GBDT) [20] is a widely used and useful algorithm that can be used for both classification and regression problems [42–47]. Recently, Ke et al. proposed a novel GBDT algorithm named LightGBM [48], which utilize two novel techniques: Gradient-based One-Side Sampling (GOSS) along with Exclusive Feature Bundling (EFB) to deal with the huge number of data samples along with massive amount of features respectively. GOSS keeps all the examples with large gradients and conducts random sampling on the examples with small gradients. EFB algorithm can bundle many exclusive characteristics to the much fewer dense characteristics, which can dramatically avoid unnecessary calculation for zero feature values. Here we apply LightGBM to build the DNA-binding residue prediction models. The detailed steps of the LightGBM algorithm is shown in Algorithm 1.


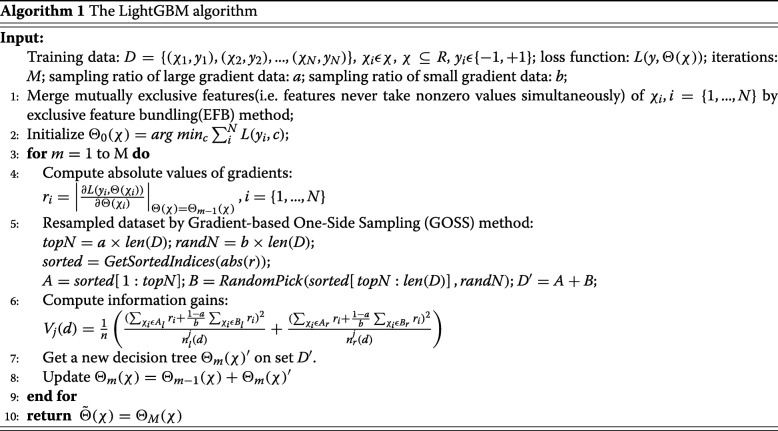


## Results

### Parameter selection

**Fig. 2:**
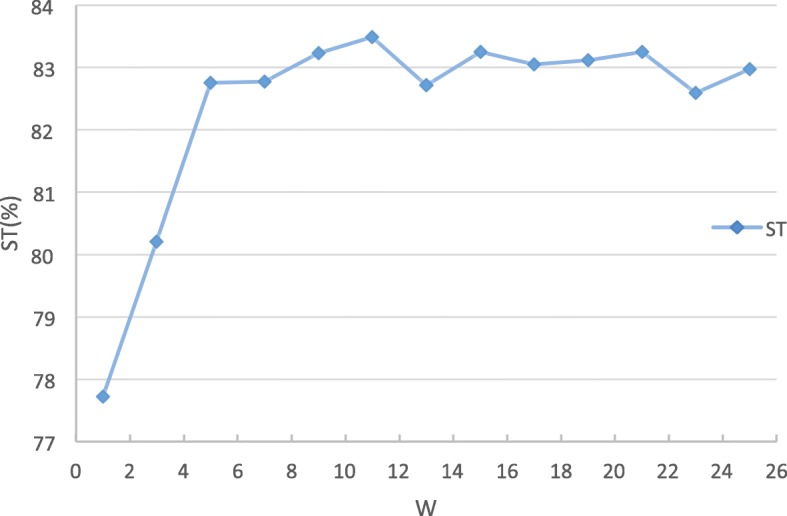
The effect of window size *w* on performance

The sliding window describes the target residue’s sequence neighborhood, and the window size *w* should be selected properly. The predictive performance of a variety of different local window sizes (1, 3,..., 25) is evaluated. As shown in Fig. 2, the ST score increases when the window size increases from 1 to 11, and the highest ST score is achieved when the window size is 11. So we select the optimal window size as 11 in the proposed PDRLGB method.

**Fig. 3:**
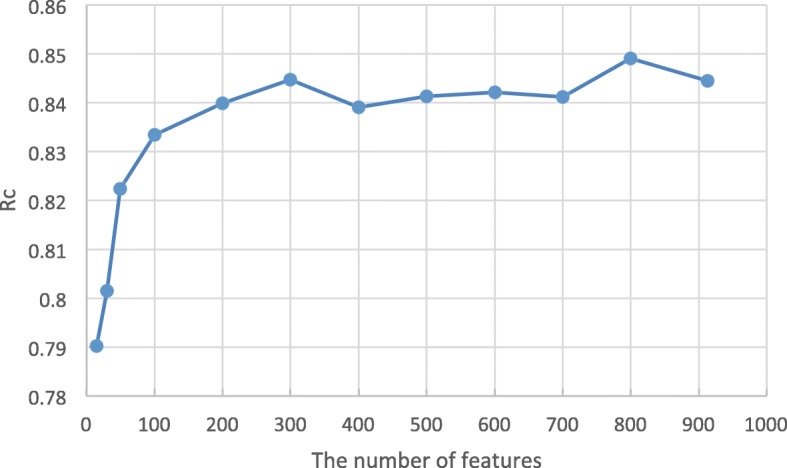
The *R*_*c*_ values of *top*−*k* feature sets obtained by using the LightGBM algorithm

The number of features (*k*) is another important parameter. We build LightGBM classifiers for each *top*−*k* subset and calculate the performance of 5-fold cross-validation. The results are shown in Fig. 3. As the dimension of the features increases, the highest RC score of 0.85 is obtained when using the top 800 features. Finally, we select a subset of features (Top 800) that contribute the most to the classification as the optimal feature set.

### Performance comparison with other machine learning techniques

**Fig. 4:**
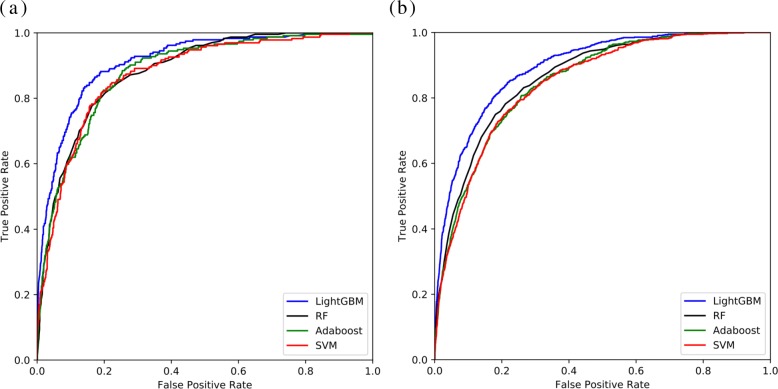
Performanc of LightGBM, SVM, Random Forest and Adaboost on the benchmark datasets. **a** shows the ROC curves on the PDNA-62 dataset. **b** shows the ROC curves on the PDNA-224 dataset

In this section, we conduct a comparison experiment of LightGBM with existing machine learning techniques, including Support Vector Machine (SVM) [49], Random Forest (RF) [40] and AdaBoost [50]. The performance of these classifiers are listed in Table 2. It is worth emphasizing that these classifiers are trained on the same benchmark with the same feature set. The ROC curves are shown in Fig. 4. It is obvious that LightGBM achieves significant performance improvement on both PDNA-62 and PDNA-224 when it compares to these classifiers. Concretely, on the PDNA-62 dataset, LightGBM obtains at least 3.1% increase on ST, 2.2% increase on F1, 3.7% increase on MCC and 2.9% increase on AUC when comparing with SVM, RF and AdaBoost. As for the PDNA-224 dataset, LightGBM achieves at least 3.1% increase on ST, 0.6% increase on F1, 3.2% increase on MCC and 3.0% increase on AUC. Due to the imbalanced problem on both datasets, the ROC curve is regarded as the useful estimation for the overall performance. Higher ROC curve denotes better prediction performance. Figure 4a and b also show that LightGBM obtains the best ROC curves on the two datasets (PDNA-62 and PDNA-224). The results imply that the LightGBM algorithm we used is more superior than other widely used classifiers.

**Table 2:** Performance comparison of LightGBM with other machine learning methods

Dataset	Methods	ACC	SN	SP	ST	PRE	F1	MCC	AUC
PDNA-62	SVM	0.817	0.745	0.829	0.787	0.433	0.547	0.468	0.873
	Adaboost	0.814	0.791	0.818	0.804	0.431	0.558	0.485	0.881
	RF	0.817	0.782	0.822	0.802	0.435	0.559	0.486	0.883
	LightGBM	0.815	0.863	0.806	0.835	0.438	0.581	0.523	0.912
PDNA-224	SVM	0.786	0.765	0.776	0.771	0.194	0.310	0.306	0.847
	Adaboost	0.773	0.761	0.774	0.768	0.192	0.307	0.320	0.851
	RF	0.814	0.750	0.819	0.784	0.226	0.347	0.351	0.864
	LightGBM	0.800	0.833	0.797	0.815	0.224	0.353	0.383	0.896

### Performance comparison with other state-of-the-art predictors

There exists many DNA-binding site prediction methods which trained and tested either on PDNA-62 or PDNA-224, such as Dps-pred [9], Dbs-pssm [51], BindN [10], Dp-bind [52], BindN-RF [13], BindN+ [53], PreDNA [19] and EL_PSSM-RT [23]. Note that some of these methods are only trained and tested the PDNA-62 dataset, and others are trained and tested on the two datasets. We calculate *P*-values using the two-tailed, paired t-test [54]. The prediction performance of our PDRLGB approach and other methods on PDNA-62 and PDNA-224 are shown in Tables 3 and 4, respectively. The results on PDNA-62 are shown in Table 3, PDRLGB achieves the best performance, outperforming other approaches by 0.9%-21.4% on ST, 1.2%-25.1% on F1, 1.6%-33.2% on MCC and 1.1%-16% on AUC. The results on the PDNA-224 dataset are shown in Table 4, PDRLGB performs better than PreDNA and EL_PSSM-RT by 2.7%-6.9% on ST, 2.9%-4.8% on F1, 4.2%-9.4% on MCC and 3.1% on AUC. These enhancements on performance indicate that the LightGBM-based PDRLGB method based on the optimally selected features is beneficial for predicting DNA-binding residues.

**Table 3:** Performance comparison of various prediction methods on PDNA-62 with 5-fold cross-validation

Methods	ACC	SN	SP	ST	PRE	F1	MCC	AUC	*P*-value
Dps-pred	0.791	0.403	0.818	0.611	0.279	0.330	0.191	-	-
Dbs-pssm	0.664	0.682	0.660	0.671	0.210	0.376	0.249	-	-
BindN	0.703	0.694	0.705	0.700	0.291	0.410	0.297	0.752	-
Dp-bind	0.781	0.792	0.772	0.782	0.378	0.512	0.490	-	-
BindN-RF	0.782	0.781	0.782	0.782	0.385	0.516	0.436	0.861	-
BindN+	0.790	0.773	0.793	0.783	0.395	0.523	0.443	0.859	-
PreDNA	0.794	0.768	0.797	0.783	0.398	0.524	0.424	-	-
EL _PSSM-RT	0.808	0.854	0.801	0.826	0.428	0.569	0.507	0.901	**
PDRLGB	0.815	0.863	0.806	0.835	0.438	0.581	0.523	0.912	1.79 ×10^−5^

**Table 4:** Performance of PDRLGB Compared with PreDNA and EL _PSSM-RT on PDNA-224 with 5-fold cross-validation

Methods	ACC	SN	SP	ST	PRE	F1	MCC	AUC	*P*-value
PreDNA	0.791	0.695	0.798	0.746	0.195	0.305	0.289	-	-
EL _PSSM-RT	0.781	0.796	0.780	0.788	0.203	0.324	0.341	0.865	**
PDRLGB	0.800	0.833	0.797	0.815	0.224	0.353	0.383	0.896	4.07 ×10^−5^

### Performance comparison on the independent test dataset

**Fig. 5:**
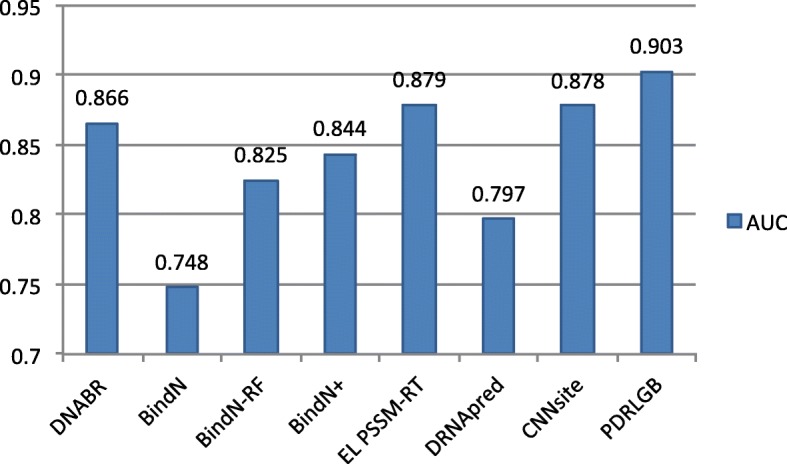
Performance comparison on TS72

To further assess the performance, we compare PDRLGB with seven existing state-of-the-art protein-DNA binding site prediction methods, DNABR [22], BindN [10], BindN-RF [13], BindN+ [53], EL_PSSM-RT [23], DRNApred [55] and CNNsite [56] on the TS-72 dataset. DNABR [22] and BindN-RF [13] are built using random forest (RF). BindN [10] and BindN+ [53] are trained using support vector machines (SVMs). EL_PSSM-RT [23] is built using a ensemble learning classifier. DRNApred [55] is designed by using a two-layer predictor, which integrats hidden Markov model (HMM) and logistic regression models. CNNsite [56] is built using Convolutional Neural Network. The AUC scores of these approaches are shown in Fig. 5. DNABR, BindN, BindN-RF, BindN+, EL_PSSM-RT, DRNApred and CNNsite achieve AUC values of 0.866, 0.748, 0.825, 0.844, 0.879, 0.797 and 0.878, respectively. Comparing with these methods, our PDRLGB approach achives the highest AUC value of 0.903 and improves the AUC score by 2.4%-15.5% on the independent dataset TS-72.

We also compare our PDRLGB method with DP-Bind [57], EL_PSSM-RT [23] and DRNApred [55] on the independent dataset TS-61. DP-Bind is implemented using machine learning algorithms including SVM, kernel logistic regression and penalized logistic regression. DP-Bind also implements two ensemble classifiers by using majority voting (MAJ) and unanimity voting (STR) respectively. Here we only compare with DP-Bind (STR) since the unanimity voting approach achieves the best performance according to Hwang *et al* [57]. The results are depicted in Table 5. We observe that PDRLGB gains the highest AUC score of 0.850. Although DRNApred has the highest specificity, PDRLGB has a better balance between recall and specificity.

**Table 5:** Performance comparison on TS-61

Methods	ACC	SN	SP	ST	PRE	F1	MCC	AUC	*P*-value
DP-Bind(STR)	0.802	0.687	0.811	0.749	0.229	0.344	0.315	-	-
DRNApred	0.772	0.337	0.966	0.652	0.450	0.386	0.347	0.822	-
EL _PSSM-RT	0.773	0.726	0.777	0.752	0.211	0.327	0.305	0.839	**
PDRLGB	0.807	0.694	0.817	0.758	0.237	0.353	0.325	0.850	7.83 ×10^−5^

### Computing time comparison

**Fig. 6:**
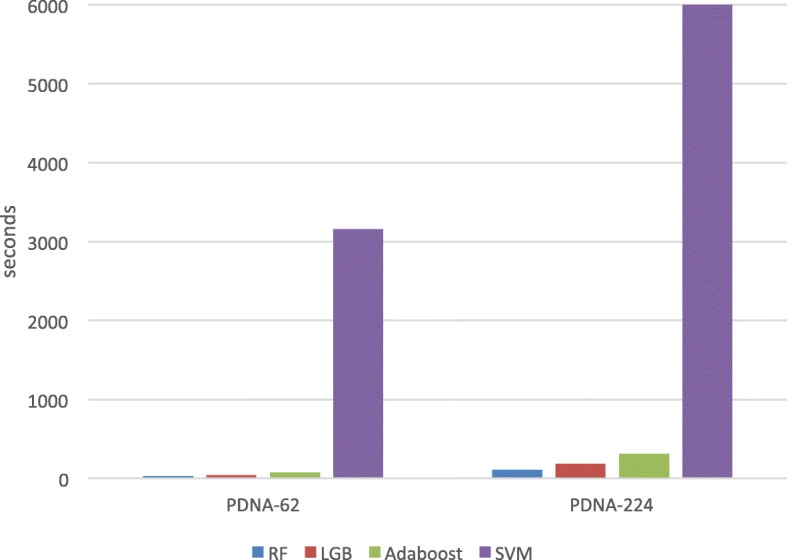
Training time of LightGBM, SVM, Random Forest and Adaboost

We present the training time cost comparisons in this subsection, which is shown in Fig. 6. Our experiments on the two datasets show that LightGBM speeds up the training process of classical methods by up to over 20 times faster than SVM and is also faster than Adaboost. Although random forest (RF) and LightGBM have similar calculation speed, in fact, the performance of the LightGBM-based method is far better than that of the RF classifier. Therefore, the PDRLGB is an accurate and fast model in the prediction of protein-DNA binding residues in the protein.

### Case study

**Fig. 7:**
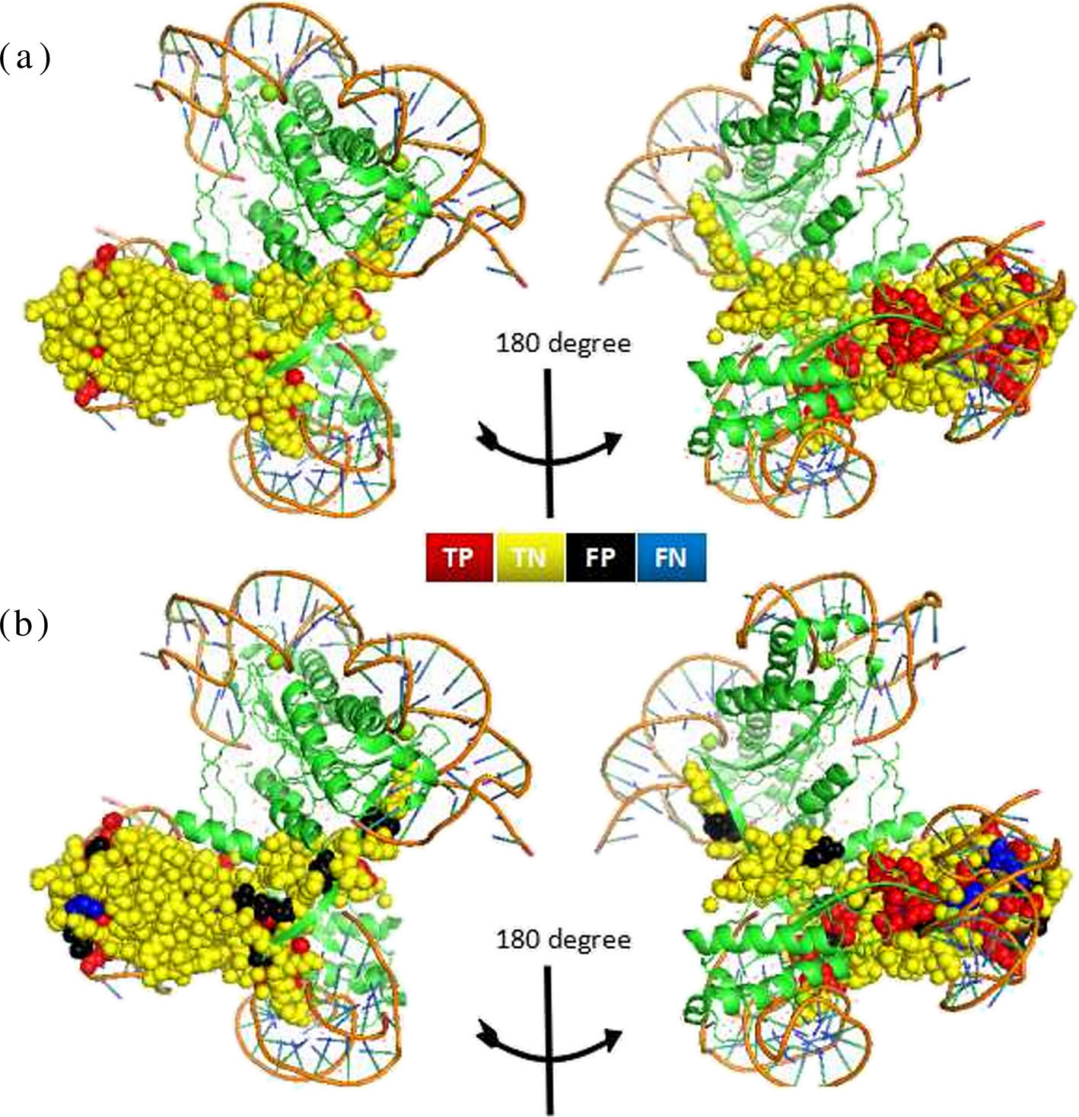
Prediction results on the case study 2XMA. **a** shows the experimentally determined DNA-binding residues in protein 2XMA:A. **b** shows the predicted binding sites by PDRLGB, and the numbers of predicted TP, FP, TN and FN in 2XMA:A are 26, 14, 95, and 4, respectively. The true positive (TP), true negative (TN), false positive (FP) and false negative (FN) sites are displayed in red, yellow, black and blue, respectively

In order to further validate the usefulness of PDRLGB for DNA-binding residue prediction, we apply PDRLGB trained on PDNA-62 to distinguish the binding residues from non-binding residues for the ISDra2 transposase/IS end complex which is not in the training set, namely, 2XMA [58]. Here, we use PDRLGB to investigate the DNA-binding residues (2XMA:A). PDRLGB achieves 87.05% on ACC, 0.67 on MCC, 86.67% on SP, 87.16% on SN, 86.91% on ST, which is very precise when compared with the available experimental data in the PDB database. The experimentally determined DNA-binding sites and predicted sites by PDRLGB for complex 2XMA are shown in Fig. 7. Figure 7a denotes the experimentally determined binding sites of protein 2XMA:A and the red spheres represent real DNA-binding sites. Figure 7b presents the predicting binding sites of protein 2XMA:A. The results show that the majority of the DNA-binding residues are correctly predicted by the PDRLGB model.

## Discussion

Existing methods for predicting DNA-binding sites are mainly divided into sequence-based methods, structure-based methods and hybrid methods. In this study, we integrate both sequence and structural features to effectively predict DNA-binding residues. A limitation of our PDRLGB approach is that it requires the protein structural information, which may limit its application. However, with the increasing solved protein structures, protein homology modeling projects and predicted 3D structures, it is expected that PDRLGB can be used as a powerful tool to effectively identify DNA-binding residues. We believe that PDRLGB can be an effective tool for accurately predicting DNA-binding residues with the increasing availability of high-quality protein-DNA complex structures.

## Conclusion

Targeting specific DNA-binding amino acids that contribute to the strength and specificity of protein-DNA interactions has broad applications ranging from rational drug design to the investigation of metabolic and signal transduction networks. In this paper, we have developed a novel LightGBM-based algorithm termed PDRLGB, for DNA-binding residue prediction. The sequence features and structural characteristics are combined to construct the feature space, and random forest combined with incremental feature selection is applied to make a feature selection. As a result, the prediction performance on the two datasets PDNA-62 and PDNA-224 with five-fold cross-validation demonstrate that PDRLGB can accelerate the training process and performs better when compared with other widely used machine learning classifiers. At the same time, performance comparisons between PDRLGB and other existing state-of-the-art DNA-binding site prediction methods demonstrate that our PDRLGB approach achieves the best performance. We have also employed our PDRLGB to identify binding sites on a protein-DNA complex 2XMA and obtained satisfactory results.
